# Executive Control Deficits Potentiate the Effect of Maladaptive Metacognitive Beliefs on Posttraumatic Stress Symptoms

**DOI:** 10.3389/fpsyg.2018.01898

**Published:** 2018-10-08

**Authors:** Joseph R. Bardeen, Thomas A. Fergus

**Affiliations:** ^1^Department of Psychology, Auburn University, Auburn, AL, United States; ^2^Baylor Psychology and Neuroscience Department, Baylor University, Waco, TX, United States

**Keywords:** metacognition, posttraumatic stress, trauma, cognitive control, executive control

## Abstract

The metacognitive model and recent preliminary research suggests that metacognitive beliefs (i.e., beliefs about thinking) may be particularly important for understanding the pathogenesis of posttraumatic stress (PTS). The metacognitive model also suggests that deficits in executive control (i.e., metacognitive control) may increase the impact of metacognitive beliefs on PTS symptoms. Trauma-exposed adult participants (*N* = 469), recruited through an online crowdsourcing website, completed a battery of measures assessing the constructs of interest. As predicted, deficits in executive control strengthened the positive association between metacognitive beliefs and PTS symptoms. This effect was found in relation to positive (e.g., “Worrying will keep me safe”), but not negative (e.g., “My thoughts are uncontrollable”), metacognitive beliefs. Supplemental analyses, indicated that the interaction between positive metacognitive beliefs and executive control significantly predicted all PTS cluster scores (i.e., Intrusion, Cognition, Arousal, Avoidance). Taken together, results support the proposal that executive control deficits potentiate the effect of metacognitive beliefs on PTS symptoms. Intervention strategies designed to strengthen executive control (e.g., the attention training technique) may be useful in treating individuals with PTS.

## Introduction

A large majority of the U.S. population will be exposed to one or more traumatic events at some point in the lifespan (Breslau, 2009), and approximately 6 to 8% of the U.S. population (i.e., Kessler et al., 2005; Kilpatrick et al., 2013), as well as 5–20% of returning military personnel (Ramchand et al., 2010), will develop posttraumatic stress disorder (PTSD; *Diagnostic and Statistical Manual of Mental Disorders* [*DSM-5*]; American Psychiatric Association [APA], 2013) following trauma exposure. Cognitive-behavioral therapy (CBT) and its underlying theoretical model has resulted in advances to our understanding and treatment of PTSD, thereby aiding in the reduction of the substantial personal and societal burden associated with PTSD (Amaya-Jackson et al., 1999; Brady et al., 2000). Nevertheless, approximately 41% of individuals with PTSD will be classified as non-responders following CBT (McDonagh et al., 2005), and this number may be as high as 72% in community clinical settings (Zayfert et al., 2005).

Whereas cognitive-behavioral models of PTSD suggest that the content of one’s thoughts (e.g., beliefs about the self, others, and the world) play a central role in conceptualizing and treating PTSD (e.g., Foa and Rothbaum, 1998; Ehlers and Clark, 2000), the metacognitive model posits that PTSD develops as a function of one’s beliefs about thinking (i.e., metacognitive beliefs), and subsequent maladaptive coping (i.e., the cognitive attentional syndrome [CAS]), rather than content-based cognitive themes (Wells and Sembi, 2004; Wells, 2009). Consistent with this conceptualization of PTSD, empirical evidence suggests that metacognitive beliefs may be more important than trauma-related thought content in the pathogenesis of PTS. For example, Fergus and Bardeen (2017a) found that associations between content-specific beliefs and PTS symptoms were attenuated or rendered non-significant after accounting for metacognitive beliefs in a sample trauma-exposed community adults (*N* = 299).

Within the metacognitive model of PTSD (Wells and Sembi, 2004; Wells, 2009), PTS symptoms are viewed as a normative part of an adaptation process in the acute aftermath of trauma exposure. The CAS (i.e., heightened self-focused attention and threat monitoring, as well as use of rumination, worry, or other avoidant coping strategies), which is initiated and maintained by metacognitive beliefs, is thought to account for the duration and severity of PTS symptoms following trauma exposure. Positive metacognitive beliefs (e.g., “Worrying helps me to avoid problems in the future”) are thought to lead to the use of the CAS following trauma exposure. Positive metacognitive beliefs are strengthened when feared outcomes do not occur. Negative metacognitive beliefs surrounding the uncontrollability and danger of thoughts (e.g., “If I could not control my thoughts, I would not be able to function”) are thought to increase attention toward internal experience (e.g., monitoring of thought content) and the likelihood that thought processes will be perceived as distressing. Negative metacognitive beliefs are maintained and strengthened because avoidant coping increases vigilance toward internal experience and PTS symptoms, thereby strengthening beliefs about the uncontrollability and danger of thoughts.

Despite promising preliminary findings that metacognitive beliefs may be more relevant to PTS symptoms than content-specific beliefs (Fergus and Bardeen, 2017a), relatively few studies have reported associations between metacognitive beliefs and PTS symptoms and findings have been mixed. In two studies, significant positive associations that were medium to large in size were reported between metacognitive beliefs and PTS symptoms (Roussis and Wells, 2006; Fergus and Bardeen, 2017a). In contrast, Bennett and Wells (2010) did not observe an association between positive metacognitive beliefs and PTS symptoms (*r* = -0.01), while a medium-large sized association was observed between negative metacognitive beliefs and PTS symptoms (*r* = 0.41). These discrepancies may be the result of using different measures of metacognitive beliefs. Specifically, the measure used by Bennett and Wells (2010) focuses on metacognitive beliefs related to memory, the measure used by Fergus and Bardeen (2017a) assesses metacognitive beliefs broadly, but in relation to trauma, and the measure used by Roussis and Wells (2006) broadly assesses metacognitive beliefs without on an emphasis on trauma. Apparent discrepancies in these associations may also be a function of failing to account for a third variable (i.e., moderator variable) that alters the strength of the relationship between PTS symptoms and metacognitive beliefs. A moderator impacts the strength of the relation between two other variables and can help explicate under what conditions the two variables relate to one another (Hayes, 2018). As described below, executive control (i.e., metacognitive control: Wells and Matthews, 1996; Wells, 2009) may be one such potential moderator that helps explicate when metacognitive beliefs relate to PTS symptoms.

Executive control relies on of a variety of top-down cognitive abilities that are associated with activation in the prefrontal cortex (e.g., inhibition, set shifting, working memory updating, error detection, and strategy formulation; Fernandez-Duque et al., 2000). Within the metacognitive model, the excessive conceptual processing that characterizes the CAS is thought to be exacerbated by deficits in executive control that reduce the likelihood that one can effectively disengage from internal experience (e.g., worry, rumination, and other forms of self-focused attentional processes) and maintain attentional focus on adaptive goal-relevant pursuits (i.e., value-driven behavior). The importance of considering deficits in executive control when conceptualizing PTSD from a metacognitive perspective is highlighted by the fact that a technique was developed to specifically address these deficits in metacognitive therapy. Specifically, the attention training technique (Wells, 1990) was developed to strengthen executive control processes that can be used to interrupt the excessive self-focused, threat-based processing that characterizes the CAS (Wells, 2009). Despite the conceptual importance of executive control to the metacognitive model, the impact of this construct on the relationship between metacognitive beliefs and PTS symptoms has yet to be empirically examined.

## Present Study

The purpose of the present study was to examine executive control as a moderator of the relationship between metacognitive beliefs and PTS symptoms in a trauma-exposed sample of adults. Following from the empirical evidence described above, we predicted that both positive and negative maladaptive metacognitive beliefs would be associated with PTS symptoms, and the association between negative metacognitive beliefs and PTS symptoms would be the largest in magnitude (Roussis and Wells, 2006; Fergus and Bardeen, 2017a). Additionally, based on evidence showing that individuals with PTSD exhibit relative deficits in the cognitive abilities associated with executive control (Deppermann et al., 2014; Scott et al., 2015), we predicted that executive control deficits would be positively associated with PTS symptoms. Importantly, based on metacognitive theory (Wells, 2009), which suggests that CAS-based coping is exacerbated by deficits in executive control, we predicted that the magnitude of the positive association between maladaptive metacognitive beliefs (i.e., positive and negative) and PTS symptoms would become significantly stronger as deficits in executive control increased. Finally, we conducted an exploratory analysis examining significant domain-specific interaction effects in the context of PTS cluster scores (i.e., clusters B [Intrusion], C [Avoidance], D [Cognition], and E [Arousal]: *DSM-5* PTSD, American Psychiatric Association [APA], 2013). Given the exploratory nature of these analyses, no *a priori* hypotheses were made.

## Materials and Methods

### Participants and Procedure

A total of 597 adults were recruited via Amazon Mechanical Turk (MTurk). MTurk is an online labor market where adults from the general population can be recruited to complete questionnaires in exchange for payment. MTurk samples tend to be more demographically diverse than American undergraduate samples (Buhrmester et al., 2011) and a number of studies support the quality of data collected via MTurk (e.g., Behrend et al., 2011; Buhrmester et al., 2011; Shapiro et al., 2013; Paolacci and Chandler, 2014). Recruitment was limited to MTurk users located within the United States and between the ages of 18–65. Additionally, to be included in the present study, participants had to report exposure to a traumatic event (Criterion A: exposure to actual or threatened death, serious injury, or sexual violence) as defined in the *DSM-5* (American Psychiatric Association [APA], 2013). The final sample (*n* = 469) consisted of adults who had experience at least one traumatic event. The average age of the final sample was 35.9 years (*SD* = 11.0) and the majority were female (61.4%). In regard to race and ethnicity, 83.6% self-identified as White, 7.0% as Black, 6.6% as Asian, 1.7% as American Indian or Alaska Native, 1.1% endorsed “other,” and 7.2% of the final sample identified their ethnicity as Hispanic.

A secure online survey program was used to administer informed consent and self-report measures. Participants were informed (via the electronic consent form) of the costs/benefits of study participation, that their responses were confidential, and that they were free to withdraw from the study at any time. After reading the consent form, participants were able to consent to, or opt out of, continued participation by clicking on one of two radio buttons that offered these choices. Upon study completion, participants were debriefed and paid in full. Participants were compensated $1.50 for completing study questionnaires, an amount consistent with precedence for paying MTurk workers in similar studies (Buhrmester et al., 2011). This study was approved by the local university-based institutional review board.

### Measures

#### Metacognitive Questionnaire-30 (MCQ-30)

The MCQ-30 (Wells and Cartwright-Hatton, 2004) is a 30-item measure inclusive of positive metacognitive beliefs about CAS-based coping (e.g., “worrying helps me to avoid problems in the future”) and negative metacognitive beliefs about uncontrollability and danger of thinking (e.g., “my worrying is dangerous for me”). Items of the MCQ-30 are rated on a 4-point scale ranging from 1 (*do not agree*) to 4 (*agree very much*). Higher scores indicate higher levels of maladaptive metacognitive beliefs. The MCQ-30 has exhibited adequate psychometric properties, including internal consistency, retest reliability, and construct validity (Wells and Cartwright-Hatton, 2004; Spada et al., 2008). Additionally, factor analytic results support use of a total score and subscale scores, and measurement invariance has been observed between men and women (Fergus and Bardeen, 2017b). Internal consistency of the positive and negative MCQ-30 scales was adequate in the present study (α = 0.92 and 0.91, respectively).

#### Barkley Deficits in Executive Functioning Scale-Short Form (BDEFS-SF)

The BDEFS-SF (Barkley, 2011) is a 20-item self-report measure designed to identify deficits in executive functioning. Participants are asked to use a 4-point scale (1 = *never or rarely* to 4 = *very often*) to indicate how often they exhibit behaviors associated with daily activities that are indicative of executive functioning deficits across five domains (i.e., time management, organization and problem solving, self-restraint, self-motivation, and self-regulation of emotions). The BDEFS has exhibited adequate psychometric properties in previous research, including evidence of internal consistency (Feldman et al., 2013) and criterion-related validity in relation to both self-report (e.g., Attention Deficit/Hyperactivity Disorder; Gray et al., 2014) and performance-based measures (e.g., working memory; Gray et al., 2015). Internal consistency of the BDEFS-SF total score was adequate in the present study (α = 0.95).

#### Life Events Checklist for DSM-5 (LEC-5) Extended Version

The LEC-5 (Weathers et al., 2013a) assesses exposure to 17 potentially traumatic events (e.g., sexual assault, motor vehicle accident, and combat). For each event, respondents are asked to indicate whether the event happened to them, they witnessed it, they learned about it, it was part of their job, they are unsure, or the event did not apply to them. For the extended version of the LEC-5, participants are asked to provide a brief narrative of the events endorsed on the screening page. They then answer a series of follow-up questions designed to clarify whether the endorsed events meet Criterion A (e.g., exposure to actual or threatened death, serious injury, or sexual violence; American Psychiatric Association [APA], 2013).

#### PTSD Checklist for DSM5-Civilian Version (PCL-5)

The PCL-5 (Weathers et al., 2013b) is a 20-item self-report measure designed to assess symptoms in clusters B (Intrusion), C (Avoidance), D (Cognition), and E (Arousal) of the *DSM-5* PTSD criteria (American Psychiatric Association [APA], 2013). Participants were asked to rate how much they have been bothered by each symptom in the past month (0 = *not at all* to 4 = *extremely*), with higher scores indicating greater PTS symptoms. Cluster scores were calculated by summing ratings for each item within a particular symptom cluster. Consistent with evidence suggesting that PTSD is a dimensional construct rather than a discrete clinical syndrome (e.g., Ruscio et al., 2002; Forbes et al., 2005; Broman-Fulks et al., 2006), items were summed to create both total and cluster scores. The PCL-5 has demonstrated adequate psychometric properties, including internal consistency, retest reliability over a 1-week period, and convergent and discriminant validity (Blevins et al., 2015). Internal consistency of the total score and subscale scores was adequate in the present study (i.e., total score α = 0.97, subscale scores from 0.88 to 0.93).

### Data Analytic Strategy

Meng et al. (1992) test for dependent correlations was used to test the hypothesis that the association between negative metacognitive beliefs and PTS symptoms would be larger in magnitude than the association between positive metacognitive beliefs and PTS symptoms. Next, SPSS version 24 (SPSS IBM, New York) was used to conduct a hierarchical regression to test the hypothesized interactive effects. Consistent with Aiken and West (1991), the predictor (i.e., metacognitive beliefs) and moderator (i.e., executive functioning) variables were mean centered and interaction terms were calculated as the product of the moderator and predictor variables. The predictor variables were entered into the first step of the model (negative and positive metacognitive beliefs), the moderator was entered into the second step of the model (executive functioning), and the interaction terms were entered into the third step of the model (executive functioning by negative and positive metacognitive beliefs). PTS symptoms served as the outcome variable in each model. Simple slopes analysis was used to further examine significant interaction effects (Aiken and West, 1991). Simple slopes analysis helps to explicate under what conditions two variables relate to one another (Hayes, 2018). More specifically, simple slopes analysis consists of constructing two simple regression equations in which the relationship between the independent variable and the dependent variable is tested at both high (+1 *SD*) and low (-1 *SD*) levels of the moderating variable (i.e., executive functioning).

Next, interaction effects (i.e., positive and/or negative metacognitive beliefs by executive functioning) were examined in the context of PTS clusters scores. Structural equation modeling (SEM) and path analysis were used to conduct this examination, instead of standard regression analysis, because multiple outcome variables (i.e., PTS cluster scores) can be modeled simultaneously in SEM. For each of the two path models, metacognitive beliefs (i.e., positive or negative), executive functioning, and an interaction term (i.e., metacognitive beliefs by executive functioning) served as predictor variables in the model. The four PTS cluster scores served as outcome variables. Each model was tested using Amos software (Version 24; Arbuckle, 2010) and maximum likelihood estimation. All variables were modeled as manifest indicators. Fit statistics were not computed because just-identified models provide perfect fit to the data (Kline, 2016).

## Results

### Bivariate Correlations

Both positive and negative metacognitive beliefs positively correlated with PTS symptoms (see **Table 1**). As predicted, a test of dependent correlations revealed that PTS symptoms correlated significantly more strongly with negative metacognitive beliefs (*r* = 0.54, *p* < 0.001) than positive metacognitive beliefs (*r* = 0.46, *p* < 0.001, *z* = 2.07, and *p* = 0.02). Also of note, a positive association between executive functioning deficits and PTS symptoms was observed (*r* = 0.57, *p* < 0.001).

**Table 1:** Zero-order correlations, means, and standard deviations for study variables.

Variable	1	2	3	4	5	6	7	8
(1) MCQ-30 Positive	–							
(2) MCQ-30 Negative	0.49	–						
(3) BDEFS-SF total	0.44	0.61	–					
(4) PCL-5 Total	0.46	0.54	0.56	–				
(5) PCL-5 Intrusion	0.42	0.49	0.51	0.93	–			
(6) PCL-5 Avoidance	0.34	0.44	0.37	0.80	0.76	–		
(7) PCL-5 Cognition	0.44	0.52	0.56	0.95	0.83	0.72	–	
(8) PCL-5 Arousal	0.45	0.52	0.56	0.94	0.84	0.65	0.85	–
*Means*	10.51	11.69	33.54	18.80	4.83	2.71	6.17	5.10
*Standard deviations*	4.47	5.12	12.09	18.99	5.10	2.63	7.23	5.60

*n = 469 trauma-exposed adults; all ps < 0.001. MCQ-30 = Metacognitive Questionnaire-30; BDEFS-SF = Barkley Deficits in Executive Functioning Scale-Short Form; and PCL-5 = PTSD Checklist for DSM-5–Civilian Version.*

### Predicting Total Posttraumatic Stress Symptoms

An examination of scatterplots (refer to **Supplementary Figures S1**, **S2**) and the Durbin–Watson statistic indicated that the regression assumptions [i.e., additivity and linearity, independent errors (Durbin–Watson statistic = 1.85), homoscedasticity, and normally distributed errors] were met (see Cohen et al., 2003). Moreover, an examination of multivariate outliers suggested that none of the cases exhibited undue influence on the estimates within the regression model (defined as >1 *DFFITS_i_*; Cohen et al., 2003). Additionally, multicollinearity statistics were all above recommended levels (tolerance statistics >0.10 and VIF<10; Cohen et al., 2003), thus indicating no robust problems related to multicollinearity.

**FIGURE 1:**
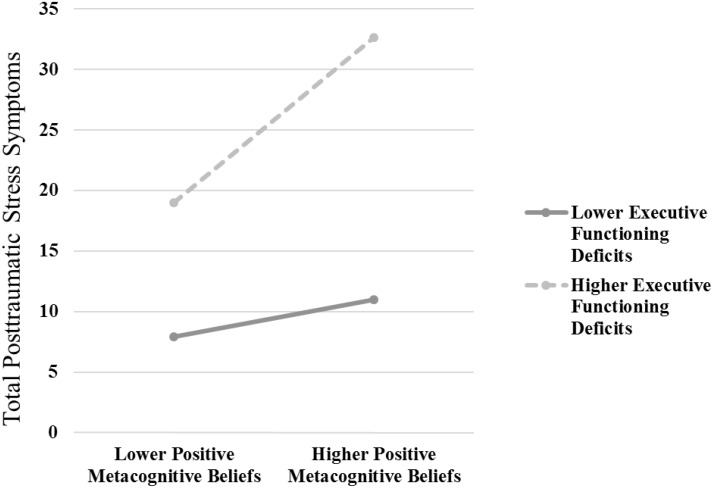
The interaction effect [positive metacognitive beliefs (assessed via the Metacognitive Questionnaire-30) by executive functioning deficits (assessed via the Barkley Deficits in Executive Functioning Scale-Short Form)] predicting total posttraumatic stress (PTS) symptoms (assessed via the PTSD Checklist for DSM5-Civilian Version). Simple slopes analysis revealed a positive association between positive metacognitive beliefs and PTS symptoms that was significant at higher (β = 0.43, *p* < 0.001), but not lower (β = 0.08, *p* = 0.18), levels of executive functioning deficits.

In the first step of the regression model (*adjusted* *R*^2^ = 0.34, *p* < 0.001), positive and negative metacognitive beliefs significantly predicted PTS symptoms (βs = 0.26 and 0.42, respectively, *p*s < 0.001). In the second step of the model (Δ*R*^2^ = 0.07, *p* < 0.001), executive functioning significantly predicted PTS symptoms (β = 0.33, *p* < 0.001). In the third step of the model (Δ*R*^2^ = 0.03, *p* < 0.001), the interaction between positive metacognitive beliefs and executive functioning significantly predicted PTS symptoms (β = 0.18, *p* < 0.001), but the interaction between negative metacognitive beliefs and executive functioning did not (β = -0.02, *p* = 0.65). The non-significant interaction term (negative metacognitive beliefs by executive functioning) was removed from the model to provide an accurate interpretation of simple effects for the significant interaction (positive metacognitive beliefs by executive functioning) in simple slopes analysis. Simple slopes analysis revealed a positive association between positive metacognitive beliefs and PTS symptoms that was significant at higher (β = 0.43, *p* < 0.001), but not lower (β = 0.08, *p* = 0.18), levels of executive functioning deficits (see **Figure 1**).

### Predicting Posttraumatic Stress Symptom Cluster Scores

**FIGURE 2:**
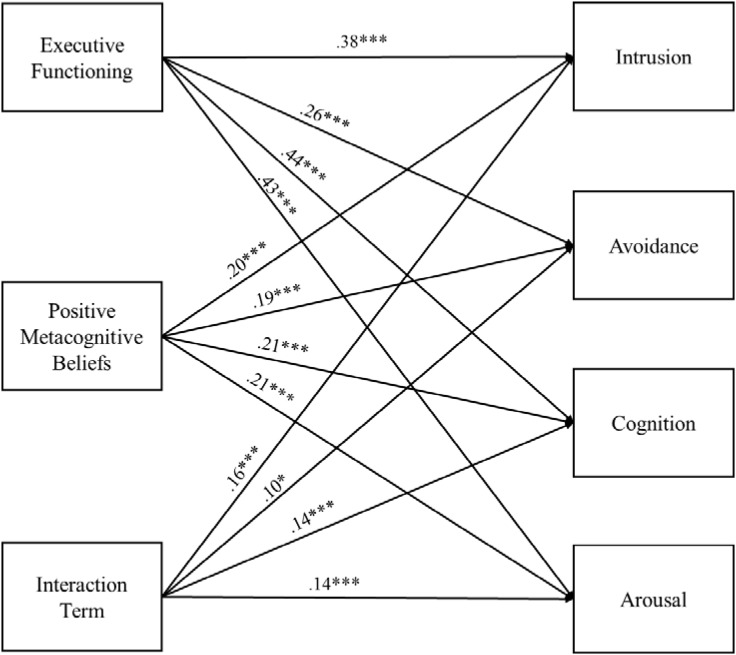
Path model with standardized path coefficients. Executive functioning = the Barkley Deficits in Executive Functioning Scale-Short Form (BDEFS-S) total score; Positive metacognitive beliefs = the Metacognitive Questionnaire-30 (MCQ-30) Positive subscale score; Interaction term = BDEFS-S × MCQ-30 Positive; Intrusion, Avoidance, Cognition, and Arousal = the four symptom clusters identified in the DSM-5 criteria for a diagnosis of PTSD (American Psychiatric Association [APA], 2013) as a assessed by the PTSD Checklist for DSM5-Civilian Version. *^∗^p* < 0.05 and *^∗∗∗^p* < 0.001.

Because the positive negative metacognitive beliefs by executive functioning interaction was significant in our primary analytic model, we conducted a path analysis in which the interaction between positive metacognitive beliefs and executive functioning predicted PTS cluster scores. Standardized path coefficients are presented in **Figure 2**. As can be seen in **Figure 2**, positive metacognitive beliefs, executive functioning, and the interaction term (positive metacognitive beliefs by executive functioning) significantly predicted each of the four PTS cluster scores (all *p*s < 0.05). Following from our primary analysis, the interaction effect was further explored using simple slopes analysis (Aiken and West, 1991). Simple slopes analysis revealed significant positive associations between positive metacognitive beliefs and each PTS cluster score at higher (Intrusion: β = 0.34, Avoidance: β = 0.28, Cognition: β = 0.34, and Arousal: β = 0.34, *p*s < 0.001), but not lower (Intrusion: β = 0.05, *p* = 0.46, Avoidance: β = 0.10, *p* = 0.14, Cognition: β = 0.08, *p* = 0.22, and Arousal: β = 0.09, *p* = 0.16), levels of executive functioning deficits.

Although the negative metacognitive beliefs by executive functioning interaction was not significant in our primary analytic model, we conducted a second path analysis in which the interaction between negative metacognitive beliefs and executive functioning predicted PTS cluster scores to ensure that the interaction terms did not exhibit significant associations with specific PTS clusters. Negative metacognitive beliefs and executive functioning significantly predicted each of the four PTS cluster scores (Intrusion: β = 0.28 and 0.32, Avoidance: β = 0.33 and 0.16, Cognition: β = 0.27 and 0.37, and Arousal: β = 0.27 and 0.37, *p*s < 0.01). Consistent with our primary analytic model, the interaction term (i.e., negative metacognitive beliefs by executive functioning) did not significantly predict any of the PTS cluster scores (Intrusion: β = 0.05, Avoidance: β = 0.03, Cognition: β = 0.07, and Arousal: β = 0.07, *p*s > 0.05).

## Discussion

As predicted, executive control deficits moderated the relationship between maladaptive metacognitive beliefs and PTS symptoms in a sample of trauma-exposed adults. Specifically, as executive control deficits increased, the strength of the association between positive metacognitive beliefs and PTS symptoms also increased. This pattern of findings is consistent with the metacognitive model (Wells, 2009), which suggests that deficits in executive control reduce the likelihood of successfully disengaging from CAS-based coping in response to internal experience (e.g., worry, rumination, and other forms of self-focused attention). These findings are also consistent with evidence that suggests that top-down executive control processes (e.g., inhibition, set shifting, working memory updating) can be used to protect those who are vulnerable to maladaptive psychological outcomes from experiencing such outcomes (Fergus et al., 2012; Jones et al., 2012; Bardeen and Fergus, 2016).

An examination of raw correlations in the present study was consistent with previous research showing that the association between negative metacognitive beliefs and PTS symptoms is larger in magnitude than the association between positive metacognitive beliefs and PTS symptoms (Roussis and Wells, 2006; Fergus and Bardeen, 2017a). However, an aggregate effect of negative metacognitive beliefs and executive control deficits on PTS symptoms was not observed. One explanation for this null result is that the relationship between negative metacognitive beliefs and PTS symptoms is more direct than the relationship between positive metacognitive beliefs and PTS symptoms. That is, the amount of time one has to enact top-down regulatory processes before distress is experienced could be shorter in duration for negative, versus positive, metacognitive beliefs. In support of the proposition, Roussis and Wells (2006) found that the association between positive metacognitive beliefs and PTS symptoms was accounted for by CAS-based coping (i.e., use of worry as a thought control strategy), whereas negative metacognitive beliefs had a direct effect on PTS symptoms independent of such coping. Put more succinctly, positive metacognitive beliefs, such as “worrying helps me to avoid problems in the future,” are likely to lead to continued processing, but do not necessarily pose an immediate threat. In contrast, negative metacognitive beliefs, such as “my worrying could make me go mad,” have a clear sense of urgency, and thus, the buffering effect of executive control may be of less benefit for those whose metacognitive beliefs are primarily about the uncontrollability and danger of thinking.

Another plausible explanation is that the diverse content of the MCQ-30 negative metacognitive beliefs subscale may be partially responsible for the null finding. The negative metacognitive beliefs subscale consists of items denoting either danger or uncontrollability. Following from the hypothesis above, executive control may have an impact on the relationship between negative metacognitive beliefs related to uncontrollability, but not danger, and PTS symptoms. Separately assessing uncontrollability and danger metacognitive beliefs in future research may be beneficial.

An exploratory aim of the present study was to examine significant domain-specific interaction effects in the context of PTS cluster scores. Results of a path analysis indicated that the interaction between positive metacognitive beliefs and executive control significantly predicted all PTS cluster scores (i.e., Intrusion, Cognition, Arousal, and Avoidance). At higher levels of executive control deficits, the magnitude of the associations between positive metacognitive beliefs and each PTS cluster score were similar in size (i.e., 0.28 to 0.34). Given its emphasis on distress associated with intrusive cognitive content, one might hypothesize that the observed interaction effect might be particularly relevant to the Intrusion cluster. However, cognitive content is present in some form for all four of the PTS symptom clusters. Trauma-related thoughts are referenced in the avoidance cluster. Memory difficulties, negative expectations about one’s self, others and the world, self- or other-blame, and other internal content make up the Cognitions cluster. And finally, the Arousal cluster references concentration difficulties, as well as hypervigilance toward perceived threat (i.e., CAS threat monitoring; *DSM-5*: American Psychiatric Association [APA], 2013).

The present results should be considered in light of study limitations. Internet samples of community adults have been used to examine trauma and PTS symptoms in prior research (e.g., Seligowski and Orcutt, 2016). Moreover, evidence supports MTurk as a viable method for collecting data for clinical research (Chandler and Shapiro, 2016) and established quality control methods were used in the present study to improve study data (e.g., using high reputation MTurk workers; Peer et al., 2014). Nonetheless, MTurk samples are not representative of the general population. As such, replicating study findings in samples with more racial/ethnic diversity, male representation, and higher levels of psychological distress (i.e., clinical samples) will be important in the future to ensure that study findings generalize. Despite utilization of a sample unselected based upon symptom severity, it is important to note that a considerable proportion of the trauma-exposed sample reported the presence of clinically relevant PTS symptoms (i.e., 29% using the more liberal PCL-5 cut score of 28 and 19.2% using the most conservative PCL-5 cut score of 37; Blevins et al., 2015).

The cross-sectional study design may also be considered a study limitation. Future research using longitudinal study designs will help clarify the temporal nature of relations among metacognitive beliefs, executive control deficits, and PTS symptoms. Additionally, experimental designs will be helpful in determining temporal precedence, as well as in determining whether executive functioning deficits are a moderator of the relationship between positive metacognitive beliefs and PTS symptoms, or vice versa. As described, executive control consists of a variety of top-down cognitive processes that are associated with activation in the prefrontal cortex (e.g., inhibition, set shifting, working memory updating, error detection, strategy formulation; Fernandez-Duque et al., 2000). The use of multiple objective measures (e.g., established behavioral assessments) to assess these cognitive processes will be important in future research to determine whether one or more of these specific processes is primarily responsible for the effects observed in the present study. Identification of the specific cognitive deficits that exacerbate the effect of metacognitive beliefs on PTS symptoms may aid in the development of a treatment for PTSD that has a narrower target.

To our knowledge, the present study is the first to provide evidence that executive control modulates the effect of metacognitive beliefs on PTS symptoms. Although evidence supports the use of metacognitive therapy for treating individuals with PTSD (Wells et al., 2015), the attention training technique (Wells, 1990, 2009) remains underutilized as a component of this larger treatment package. Results of the present study, in combination with evidence that the attention training technique reduces symptoms of emotional disorders as a standalone intervention (e.g., Fergus and Bardeen, 2016; Knowles et al., 2016), suggest that using the attention training technique to directly target executive control deficits may be an important adjunct to more established PTSD interventions. Moreover, given the applicability of the observed interaction to all four PTS symptom clusters, metacognitive therapy, including the attention training technique, may be particularly well-suited for treating individuals with PTSD.

## Ethics Statement

This study was carried out in accordance with the recommendations of the Institutional Review Board (IRB) in the Office of Research Compliance at Auburn University. The protocol was approved by the IRB at Auburn University. All participants were provided with a one page description of the study in order to make an informed choice about their participation. Participants were then given the option to participate in the study by clicking a “yes” or “‘no” button to indicate their consent. A waiver of documentation of written consent was approved by the IRB at Auburn University.

## Author Contributions

JB was involved in study conceptualization, data collection, data analysis and interpretation, and manuscript preparation. TF was involved in study conceptualization and manuscript preparation.

## Conflict of Interest Statement

The authors declare that the research was conducted in the absence of any commercial or financial relationships that could be construed as a potential conflict of interest.

## Supplementary Material

FIGURE S1 Examination of assumption of normally distributed errors.

FIGURE S2 Examination of assumption of homoscedasticity.
