# Supplementary figures and images for: Executive Control Deficits Potentiate the Effect of Maladaptive Metacognitive Beliefs on Posttraumatic Stress Symptoms
Source: Front Psychol. 2018 Oct 8;9:1898. doi: 10.3389/fpsyg.2018.01898 (PMC6186835; doi:10.3389/fpsyg.2018.01898)

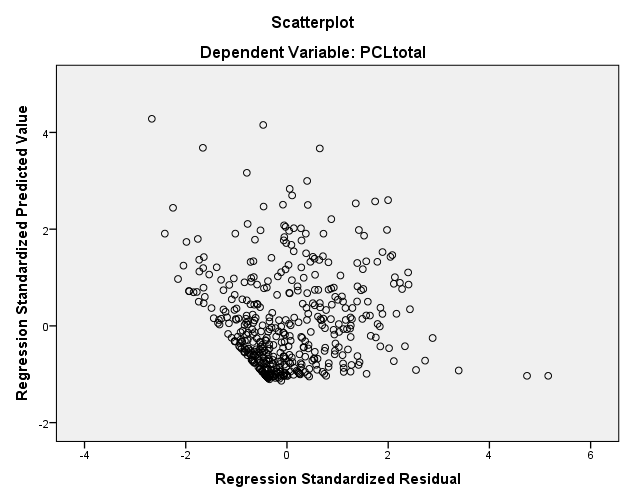

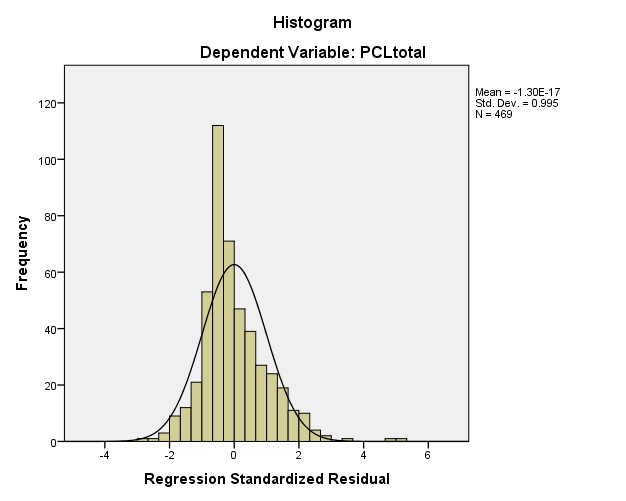

Supplement: FIGURE S1 — Examination of assumption of normally distributed errors. [file Table_1.DOCX]
